# Copper deficiency affects the developmental competence of porcine oocytes matured *in vitro*

**DOI:** 10.3389/fcell.2022.993030

**Published:** 2022-09-07

**Authors:** Hyerin Choi, Dongjin Oh, Mirae Kim, Lian Cai, Joohyeong Lee, Eunhye Kim, Gabsang Lee, Sang-Hwan Hyun

**Affiliations:** ^1^ Veterinary Medical Center and College of Veterinary Medicine, Laboratory of Veterinary Embryology and Biotechnology (VETEMBIO), Chungbuk National University, Cheongju, South Korea; ^2^ Institute of Stem Cell and Regenerative Medicine (ISCRM), Chungbuk National University, Cheongju, South Korea; ^3^ Graduate School of Veterinary Biosecurity and Protection, Chungbuk National University, Cheongju, South Korea; ^4^ Laboratory of Molecular Diagnostics and Cell Biology, College of Veterinary Medicine, Gyeongsang National University, Jinju, South Koreaa; ^5^ Department of Neurology, Institute for Cell Engineering, Johns Hopkins University School of Medicine, Baltimore, MD, United States

**Keywords:** *in vitro* maturation, porcine oocytes, embryonic development, Cu, TEPA

## Abstract

The trace element Cu is required for the activity of various enzymes essential for physiological processes. In this study, we elucidated the copper transport system in porcine follicular cells and investigated the effect of Cu chelation during *in vitro* maturation (IVM) of porcine oocytes and subsequent embryonic development after parthenogenetic activation (PA). Cu chelation was induced by adding tetraethylenepentamine (TEPA) to the maturation media (TCM199-PVA). First, we identified the localization and relative levels of the copper transporter CTR1 in follicular cells. The level of CTR1 protein was the highest in mature cumulus cells; moreover, CTR1 was mainly localized in the cytoplasmic vesicular compartment in oocytes, whereas it was evenly distributed in the cytoplasm in cumulus cells. A total of 42 h after IVM, the TEPA-treated group showed reduced maturation rates compared to those of the control (*p* < 0.05). This negative effect of TEPA disappeared when it was added to the media with Cu (Cu + TEPA group). The TEPA treatment during IVM significantly increased the mRNA levels of the *Has2* gene, which is related to cumulus expansion (*p* < 0.05). Both Cu supplementation and chelation significantly increased the reactive oxygen species (ROS) levels in porcine oocytes (*p* < 0.05). When we analyzed the transcript levels of folliculogenesis-related genes in Cu chelation conditions, only the expression of *MAPK3* in cumulus cells significantly increased compared to that of the control. We also evaluated the subsequent embryonic development of PA embryos. TEPA-treated oocytes showed significantly decreased blastocyst formation rates compared to those of the control. The TEPA-induced toxic effect was alleviated when Cu was added with TEPA. Our findings suggest that the Cu transport system plays an important role in the porcine follicular development process and that the Cu deficiency negatively affects porcine oocyte maturation, as well as their subsequent developmental competence.

## 1 Introduction

The *in vitro* maturation (IVM) of oocytes for embryo production is the most determinant step of embryo fertility and is used to improve the production of genome-modified domestic animals (Jin et al., 2018). Among species, pigs are the most similar to humans in terms of zygote metabolic reserves, the timing of embryo development to blastocyst, zygotic genome activation, and physiological characteristics (Vajta et al., 2010). Therefore, the pig is known as an ideal animal model for understanding the development of female germ cells in humans. However, despite numerous studies, the efficacy of the *in vitro* production method in pigs is still not satisfactory. Most of the porcine oocytes matured *in vitro* do not reach cytoplasmic maturity (Hyun et al., 2003). To improve the IVM rate of oocytes, various supplements, including hormones such as luteinizing hormone, human chorionic gonadotropin (hCG), pregnant mare serum gonadotropin, as well as porcine follicular fluid (pFF), antioxidants, hyaluronic acid, and amino acids (Sato et al., 1990; Stankiewicz et al., 2009; Batista et al., 2016; Yuan et al., 2017), have been added to the culture medium. However, many aspects of components in conventional oocyte maturation media have not yet been determined. In particular, pFF contains a variety of unknown factors, including growth factors, cytokines, and trace elements. These factors can influence oocyte maturation and the subsequent embryonic development (Jeon et al., 2015). Therefore, these factors or underlying mechanisms need to be determined.

Trace elements are required in small amounts, but they are essential for the normal growth and development of animals (Hostetler et al., 2003). The trace elements copper (Cu), iodine (I), iron (Fe), manganese (Mn), selenium (Se), and zinc (Zn) are known to affect embryonic survival, as well as other aspects of reproductive performance and growth in mammals (Hidiroglou, 1979; Hurley and Keen, 1987; Underwood, 1981). Especially, Cu, I, Mn, Se, and Zn appear to have the greatest impact on reproduction and have been studied most extensively (Hostetler et al., 2003). In general, trace elements are required to synthesize many proteins and activate a wide range of enzymatic systems. Metalloenzymes, which contain trace elements, participate in the process of bone formation (Leach Jr, 1967), lipid metabolism (Cunnane et al., 1993), glucose utilization (Jovanovic-Peterson and Peterson, 1996), iron transport (Raub et al., 1985), DNA synthesis (Townsend et al., 1994), and free radical metabolism (De Haan et al., 1994). Through one or more of these mechanisms, trace elements can directly affect embryonic and fetal development (Hostetler et al., 2003). Cu is an indispensable trace element during male and female reproduction (Michaluk and Kochman, 2007; Tvrda et al., 2015).

Cu plays a key role in catalytic and regulatory biochemical reactions crucial for normal growth and development (Yuan et al., 2018). Cu deficiency during pregnancy can result in early embryonic death and significant structural abnormalities, including skeletal, central nervous system, and cardiovascular defects (Prohaska, 2000). The intracellular transport of Cu is mediated by copper chaperones, such as CTR1 and Atox-1 (Grubman and White, 2014). CTR1 heterozygous null mutations result in delayed growth, open neural tubes, and embryo rotation failure, leading to death *in utero* (Kuo et al., 2001). Mechanistically, Cu deficiency affects embryonic and fetal development through multiple mechanisms, including an impaired oxidant defense system, altered angiogenesis, impaired energy production, and an altered ECM composition (Keen et al., 2003). Therefore, Cu is considered a necessary element for reproduction and embryonic development, and Cu deficiency can alter the maturation of oocytes and their subsequent developmental ability. Several studies have been conducted on the effects of Cu supplementation during IVM in bovine oocytes and have shown that Cu has an anti-apoptotic effect on cumulus cells and improves embryonic development as well as increases glutathione levels in oocytes (Picco et al., 2012; Rosa et al., 2016; Anchordoquy et al., 2022). However, the role and effects of Cu during porcine IVM have not been sufficiently studied. A previous study of our group investigated the effect of Cu supplementation during porcine IVM, which showed that 0.7 μg/ml Cu supplementation improved oocyte nuclear maturation and preimplantation embryonic development (Choi et al., 2021). In contrast, exposure to excessive Cu at 25 μg/ml leads to abnormalities in mitochondrial distribution and function, resulting in poor oocyte quality (Chen et al., 2021).

We hypothesized that a Cu transport system exists in porcine COCs, and that Cu deficiency during IVM could have a negative effect on the porcine oocyte maturation and developmental competence. In the present study, we identified the localization and relative expression of the Cu transporter CTR1 in cumulus cells and oocytes. We also investigated the effects of Cu deficiency on cumulus expansion, oocyte maturation, and subsequent developmental competence during porcine IVM using tetraethylenepentamine (TEPA), an ideal chelator for removing Cu from cells without affecting their viability or changing their phenotype (Percival and Layden-Patrice, 1992).

## 2 Materials and methods

### 2.1 Chemicals

All chemicals and reagents used in this study were purchased from Sigma-Aldrich (St. Louis, MO, United States), unless otherwise indicated.

### 2.2 Oocyte collection and *in vitro* maturation

About 100–120 porcine ovaries were obtained per day at a local slaughterhouse and transported to the laboratory within 3 h in 0.9% (w/v) NaCl saline at 37–39°C. The porcine follicular fluid (pFF) containing cumulus-oocyte complexes (COCs) was aspirated from the antral follicles (3–6 mm) using a 10-ml disposable syringe with an 18-G needle and collected into a 15 ml centrifuge tube. Following settling at 37°C for 5 min, the supernatant was removed and the precipitate was resuspended in HEPES-buffered Tyrode’s medium containing 0.05% (w/v) polyvinyl alcohol (TLH-PVA) and 300–320 COCs were recovered using a stereomicroscope. Only the COCs with three or more layers of compact cumulus cells and a homogenous cytoplasm were selected for IVM. The selected COCs were transferred to 500 µl of TCM199 (Gibco, Grand Island, NY, United States) supplemented with 0.6 mM cysteine, 0.91 mM sodium pyruvate, 10 ng/ml epidermal growth factor, 75 µg/ml kanamycin, 1 µg/ml insulin, and 0.1% (w/v) PVA. For maturation, the selected COCs were incubated for 42 h at 39°C with 5% CO_2_ and 95% air in a humidified chamber. During the entire IVM period, 0.7 µg/ml Cu or/and 25 µM TEPA were added to the maturation medium. During the first 22 h, the COCs were matured using hormones (10 IU/ml equine chorionic gonadotropin and 10 IU/ml hCG (Intervet, Boxmeer, the Netherlands). After 22 h of maturation using hormones, the COCs were cultured in the absence of eCG and hCG in a maturation medium for an additional 18–20 h.

### 2.3 Assessment of nuclear maturation

After 42 h of culture, cumulus cells were removed from the oocytes by pipetting gently with 0.1% hyaluronidase in IVM medium and the denuded oocytes were washed using TLH-PVA. The oocytes extruding first polar bodies were regarded as mature oocytes. The experiment was performed over three different days.

### 2.4 Estimation of the cumulus cell expansion index

After 42 h of IVM, the COCs from each group were visualized using an optical microscope, and images were acquired to assess the cumulus cell expansion index (CEI). The CEI was calculated using a previously described scale (Vanderhyden et al., 1990). This index ranges from 0 to 4 according to the extent of cumulus cell expansion: a score of 0 indicates no expansion; a score of 1 indicates expansion of the outermost cumulus cell layers; a score of 2 indicates expansion of the outer half of the cumulus cell layers; a score of 3 indicates expansion of all layers, except for the corona radiata; and a score of 4 indicates expansion of all layers of the cumulus cells.

### 2.5 Immunocytochemistry

*In vitro* matured oocytes were fixed using 0.1% paraformaldehyde in phosphate-buffered saline (PBS) for 30 min at RT and permeabilized using 1% Triton X-100 for 10 min. Subsequently, the oocytes were incubated in PBS/1.0% (w/v) BSA at 37°C for 1 h and then incubated overnight at 4°C with the anti-CTR1 antibody (NB100-402, Rabbit; 1:200 dilution; Novus Biologicals). After washing the oocytes three times with PBS, these were incubated at 37°C for 1 h with IgG (H+L) highly cross-absorbed goat anti-rabbit secondary antibody and Alexa Fluor^**®**^ 594 (1:400 dilution). The nuclei were stained with 10 µg/ml Hoechst 33342 and the oocytes were mounted on slides using a ProLong Gold antifade mountant (Invitrogen Corporation, Carlsbad, CA, United States). The oocytes were examined using a confocal laser microscope (Carl Zeiss, Thornwood, NY, United States), and all images were analyzed using the ZEN (blue edition) software program. Negative controls were obtained by adding PBS/1% (w/v) BSA instead of the primary antibody to the incubation medium. Cumulus cells were separated from the oocytes after the IVM and cultured on slides. Then, they were fixed and stained for CTR1, as outlined above.

### 2.6 Western blot analysis

To compare the expression level of the CTR1 protein in each cell type using western blot, porcine ovarian follicular cells, including granulosa cells, immature cumulus cells, mature cumulus cells, MI oocytes, and MII oocytes, were collected. The total protein was extracted using RIPA lysis buffer (ProEX™ CETi lysis buffer; TransLab, South Korea). The protein concentration was determined using the BCA (bicinchoninic acid) method, and the final amount of protein used for western blot was 15 μg or more. The proteins were separated using 10% SDS-PAGE gels and transferred to polyvinylidene fluoride (PVDF) membranes (Merck Millipore Corporation). The membrane was incubated at 4°C with primary antibodies (1:1,000) [β-actin rabbit monoclonal antibody (mAb) (#4967) and SLC31A1/CTR1 rabbit monoclonal antibody (ab129067)] overnight. The binding of the primary antibodies was detected using horseradish peroxidase (HRP)-conjugated anti-rabbit IgG (1:2,000) for 1 h at RT. The target proteins were detected using a Lumino graph II (ATTO, Tokyo, Japan). All experiments were performed at least three times. All antibodies used in the present study were purchased from Cell Signaling Technology, Inc. (Boston, MA, United States).

### 2.7 Gene expression analysis using quantitative real-time polymerase chain reaction

After IVM was completed, the oocytes and cumulus cells were separated from 60 COCs for each group by using 0.1% hyaluronidase to analyze the gene expression levels. Excluding the degenerated and dead oocytes, all samples were washed twice in Dulbecco’s PBS and stored at −80°C until mRNA extraction. The total RNA was extracted using TRIzol reagent (TaKaRa Bio, Inc, Otsu, Japan) and complementary DNA (cDNA) synthesis was carried out using a reverse transcription master mix (Elpis Bio, Inc., Chungcheongnam-do, Daejeon, Republic of Korea) according to the manufacturer’s instructions. To perform qRT-PCR, the synthesized cDNA (1.0 µg/µl for CCs, 0.4 µg/µl for oocytes), 2 × SYBR Premix Ex Taq (TaKaRa Bio, Inc.), and 10 pmol of specific primers (Macrogen) were added to prepare the PCR mixture. All primer sequences used in this experiment are listed in Table 1. The qRT-PCR analysis was performed using a CFX96 Touch Real-Time PCR Detection System (Bio-Rad, Hercules, CA, United States). The reactions were performed as follows: 40 cycles of denaturation at 95°C for 30 s, annealing at 58°C for 15 s, and extension at 72°C for 30 s. Relative quantification was performed using threshold cycle (Ct)-based methodologies at a constant fluorescence intensity. The relative mRNA expression (R) was calculated using the equation: $R\;=\;2^{-\left(Ct_{sample}-Ct_{control}\right)}$ . The R values obtained for each gene were normalized to those of *GAPDH* and *RN18S* in cumulus cells and oocytes, respectively.

**TABLE 1:** Primer sequences for qRT-PCR.

mRNA	Primer sequences	Product size (bp)	GenBank accession number
*RN18S*	F: 5′-CGC​GGT​TCT​ATT​TTG​TTG​GT-3′	219	NR_046261.1
	R: 5′-AGT​CGG​CAT​CGT​TTA​TGG​TC-3′		
*GAPDH*	F: 5′-GTC​GGT​TGT​GGA​TCT​GAC​CT-3′	207	NM_001206359.1
	R: 5′-TTG​ACG​AAG​TGG​TCG​TTG​AG-3′		
*Has2*	F: 5′-TTA​CAA​TCC​TCC​TGG​GTG​GT-3′	199	NM_214053.1
	R: 5′-TCA​AGC​ACC​ATG​TCG​TAC​TG-3′		
*CD44*	F: 5′-AGT​CAA​GAA​GGT​GAG​GCA​AA-3′	175	XM_021085286.1
	R: 5′-TGC​CAT​TGT​TAA​TCA​CCA​GC-3′		
*CX43*	F: 5′-ACT​GAG​CCC​CTC​CAA​AGA​CT-3′	191	NM_001244212
	R: 5′-GCT​CGG​CAC​TGT​AAT​TAG​CC-3′		
*MAPK3*	F: 5′-ATC​ACA​GTG​GAG​GAA​GCA​CT-3′	202	XM_021088019
	R: 5′-GAG​GCA​TCT​GTC​CAG​GTT​AG-3′		
*MAPK1*	F: 5′-AGT​CCA​TCG​ACA​TCT​GGT​CT-3′	240	XM_021088019
	R: 5′-GAG​CTT​TGG​AGT​CAG​CAT​TT-3′		
*Cyclin B1*	F: 5′-AGC​TAG​TGG​TGG​CTT​CAA​GG-3′	101	NM_001170768.1
	R: 5′-GCG​CCA​TGA​CTT​CCT​CTG​TA-3′		
*GDF9*	F: 5′-GGT​TCC​AGC​TTC​ATT​CAA​TC-3′	120	NM_001001909.1
	R: 5′-ACA​ATC​CAG​TTG​TCC​CAC​TT-3′		
*BMP15*	F: 5′-CCA​TCA​TCC​AGA​ACC​TTG​TC-3′	154	NM_001005155.2
	R: 5′-CAG​GAC​TGG​GCA​ATC​ATA​TC-3′		

F: Forward, R: Reverse.

### 2.8 Measurement of the intracellular reactive oxygen species and GSH levels

A total of 42 h after IVM, the MII oocytes from each group were selected to measure their intracellular ROS and GSH levels, as described previously (You et al., 2010). In brief, Cell Tracker Blue (4-chloromethyl-6.8-difluoro-7-hydroxycoumarin; Invitrogen) and 2′,7′-dichlorodihydrofluorescein diacetate (H_2_DCFDA; Invitrogen) were used to detect the intracellular GSH and ROS levels, respectively. Ten oocytes from each treatment group were incubated in the dark for 30 min in a TLH-PVA solution supplemented with 10 µM Cell Tracker or 10 µM H_2_DCFDA. The stained oocytes were washed three times with TLH-PVA and placed in a 10-µl droplet of TLH-PVA. Fluorescence was detected using an epifluorescence microscope (TE300; Nikon) with UV-2A (370 nm for GSH) and GFP-B (460 nm for ROS) filters. The images were saved in JPG format. The fluorescence intensity of the oocytes was analyzed using Adobe Photoshop CS3 Extended, and the values were normalized to those of the control group.

### 2.9 Parthenogenetic activation and *in vitro* culture of porcine embryos

After IVM, the oocytes belonging to the MII stage were selected from each group to perform the PA experiments. The collected oocytes were washed twice in a 280 mM mannitol solution containing 0.01 mM CaCl_2_ and 0.05 mM MgCl_2_. Then, the oocytes were loaded on the activation solution (a 260 mM mannitol solution supplemented with 0.001 mM CaCl_2_ and 0.05 Mm MgCl_2_) between the electrodes of the chamber. The chamber was connected to an electrical pulsing machine (LF101; Nepa Gene, Chiba, Japan) and the oocytes in the activation solution were activated with two pulses of 120 V/mm of DC for 60 µs. The electrically activated oocytes from each group were transferred into an IVC medium containing 5 µg/ml cytochalasin B and incubated in a 39°C humidified atmosphere of 5% CO_2_ and 5% O_2_ for 4 h. After that, the PA embryos were washed twice with IVC medium (porcine zygote medium 3) and 10–12 activated oocytes were cultured in 25-µl droplets of fresh IVC medium covered with mineral oil. The medium was changed every 2 days.

### 2.10 Embryo quality evaluation and total cell counts

To evaluate their developmental competence, day 0 was regarded as the day when PA was performed. On day 2 after PA (48 h), the cleavage rates were evaluated using a stereomicroscope and the embryos were categorized into three groups depending on their number of cells: 2–3, 4–5, and 6–8 cells. The rate of blastocyst formation was assessed on day 7 (168 h) after PA according to the degree of expansion and hatching status: early, expanded, and hatched, as previously described (Choi et al., 2021). To count the cell number in the blastocysts on day 7, the blastocysts from each group were collected and the zona pellucida (of all of them, excluding those in the hatched state) was removed using 0.5% protease. The zona pelucida-free blastocysts were then fixed in 0.1% paraformaldehyde and stained with 5 µg/ml Hoechst 33342 for 5 min. Finally, the blastocysts were mounted on glass slides in a drop of 100% glycerol, gently squashed under a cover slip, and observed using a fluorescence microscope (TE300, Nikon) with a UV filter (370 nm) at a ×400 magnification. Then, the cells were counted.

### 2.11 Statistical analysis

Each experiment was conducted at least three times. All statistical analyses were carried out using IBM SPSS Statistics software (version 21.0; IBM Corp., Armonk, NY, United States). The relative protein levels of CTR1, the rate of polar body extrusion, the levels of intracellular GSH and ROS, the embryonic development data (e.g., the rate of cleavage and blastocyst formation, and the total blastocyst cell number), and the relative gene expression levels were analyzed using ANOVA, followed by Duncan’s multiple range test. Statistical significance was set at *p* < 0.05. All values are reported as the mean ± standard error of the mean (SEM).

## 3 Results

### 3.1 Identification of the copper transport system in porcine oocytes and cumulus cells

**FIGURE 1:**
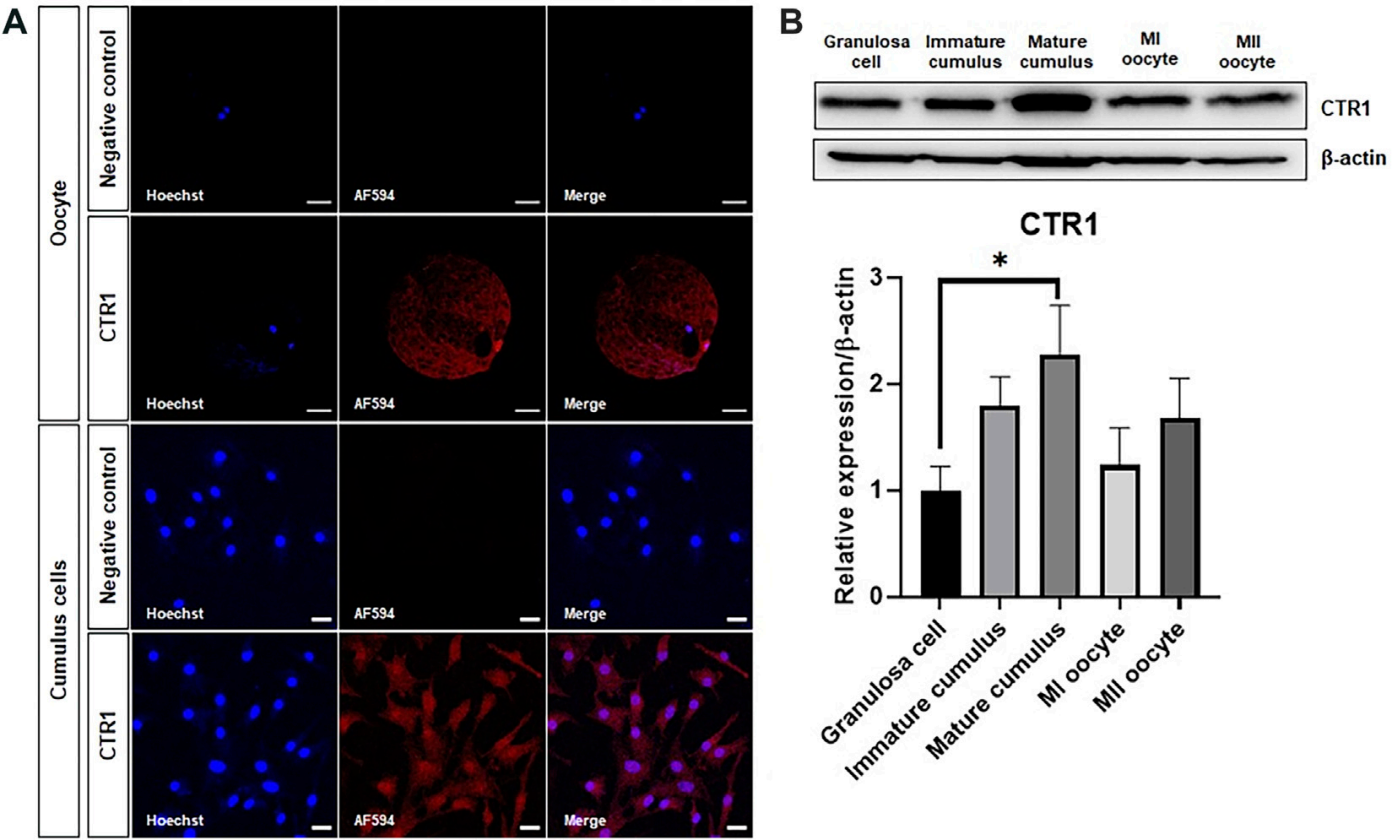
Identification of the copper transporter CTR1 in porcine oocytes and cumulus cells. **(A)** Immunofluorescence indicating the localization of the CTR1 in meiosis II stage-oocytes and mature cumulus cells. Scale bar = 50 and 20 μm, respectively. **(B)** A representative western blot image and relative densitometric bar graph of the CTR1 expression in granulosa cells, immature cumulus, mature cumulus, meiosis I (MI) oocytes, and meiosis II (MII) oocytes. An asterisk indicates statistical significance (*p < 0.05).

The purpose of this study was to confirm the localization of the CTR1 protein, known as the copper transporter, in porcine oocytes and cumulus cells. CTR1 was located in a cytoplasmic vesicular compartment concentrated around the nucleus in the oocytes, whereas it was found to be evenly distributed throughout the cytoplasm in cumulus cells (Figure 1A). Western blot was performed for quantitative comparative analysis of the CTR1 protein. The analysis exhibited that the amount of CTR1 is the highest in mature cumulus cells among granulosa cells, immature cumulus cells, mature cumulus cells, MI oocytes, and MII oocytes (Figure 1B).

### 3.2 Effect of copper chelation during *in vitro* maturation on oocyte nuclear maturation

**FIGURE 2:**
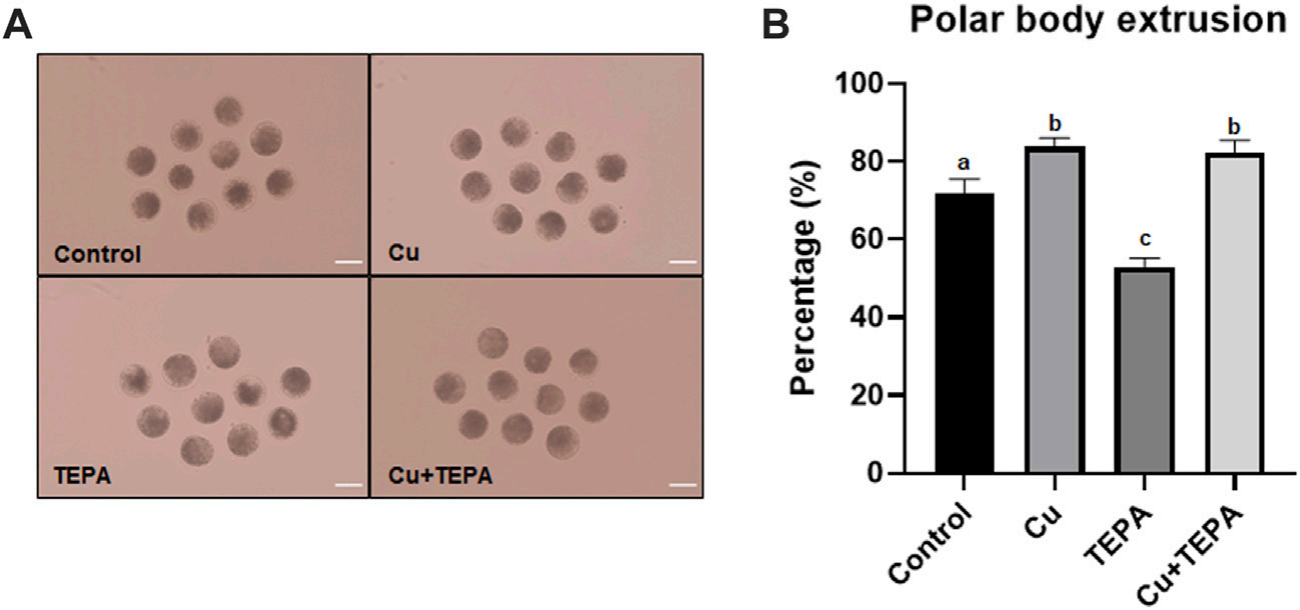
Effect of Cu chelation on the first polar body extrusion rate of *in vitro* matured oocytes. **(A)** The images of matured oocytes for each group. Sale bar = 100 μm. **(B)** A rate of polar body extrusion after Cu and TEPA supplementation during 42 h of IVM. The values represent the mean ± SEM. The values (a–c) with different superscripts within the same column are significantly different (*p* < 0.05). The experiment was replicated thrice.

To identify the effect of Cu chelation during IVM on oocyte nuclear maturation, we evaluated the percentage of oocytes showing the first polar body (Figure 2A). As shown in Figure 2B, Cu supplementation resulted in a significantly increased nuclear maturation rate (83.8 ± 1.3) compared to that of the control (71.9 ± 2.0). Conversely, the TEPA-treated oocytes showed significantly reduced maturation rates (53.0 ± 1.3). This negative effect of TEPA disappeared when it was added to media with Cu (82.4 ± 1.8, Cu + TEPA group).

### 3.3 Effect of copper chelation during *in vitro* maturation on cumulus cell expansion

**FIGURE 3:**
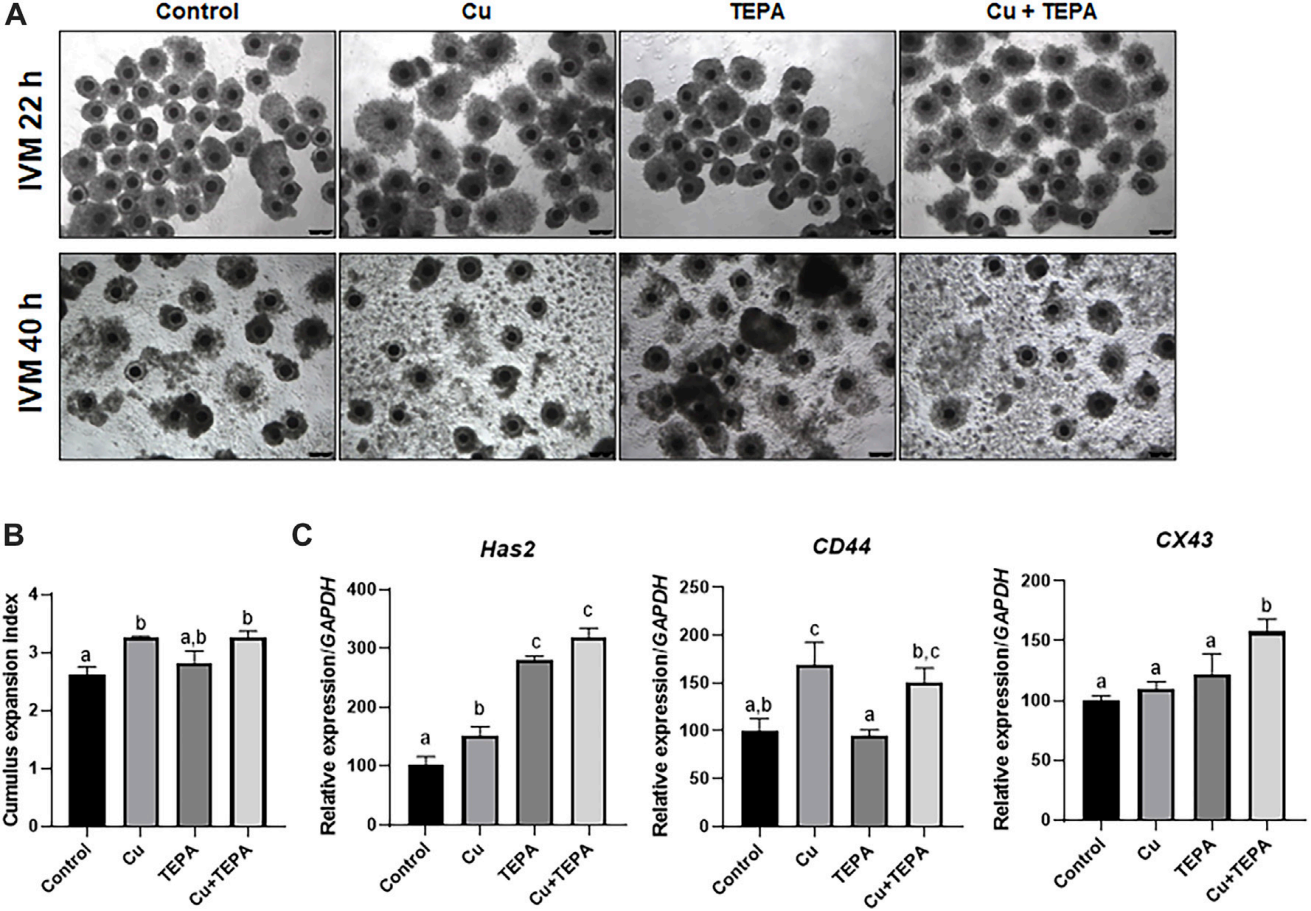
Effect of Cu chelation during *in vitro* maturation (IVM) on cumulus cell expansion in porcine COCs. **(A)** Representative morphologies of the COCs derived from each group 22 h and 40 h after IVM. Scale bars = 250 μm. **(B)** The extent of cumulus cell expansion was assessed using the subjective scoring system. **(C)** The mRNA expression levels of the cumulus cell expansion-related genes *Has2*, *CD44*, and *CX43* were compared among the cumulus cells from each group. For all graphs, the values represent the mean ± SEM. Values (a–c) with different superscripts within the same column are significantly different (*p* < 0.05). The experiment was replicated thrice.

We evaluated the effects of Cu chelation during IVM using TEPA on cumulus cell expansion. A total of 42 h after IVM, we compared the CEI and the mRNA expression levels of the related genes (Figure 3A). The TEPA-treated (3.00 ± 0.11) and the Cu-treated groups (3.23 ± 0.05) displayed a significantly increased cumulus expansion (*p* < 0.05) compared to that of the control (2.59 ± 0.15) (Figure 3B). The mRNA analysis results showed that the expression levels of *CD44* and *Has2* were significantly higher in Cu-treated cumulus cells than in the control cells (Figure 3C). TEPA supplementation only significantly increased the mRNA expression level of *Has2* (*p* < 0.05) (Figure 3C).

### 3.4 Effect of copper chelation during *in vitro* maturation on the cytoplasmic maturation of porcine oocytes

**FIGURE 4:**
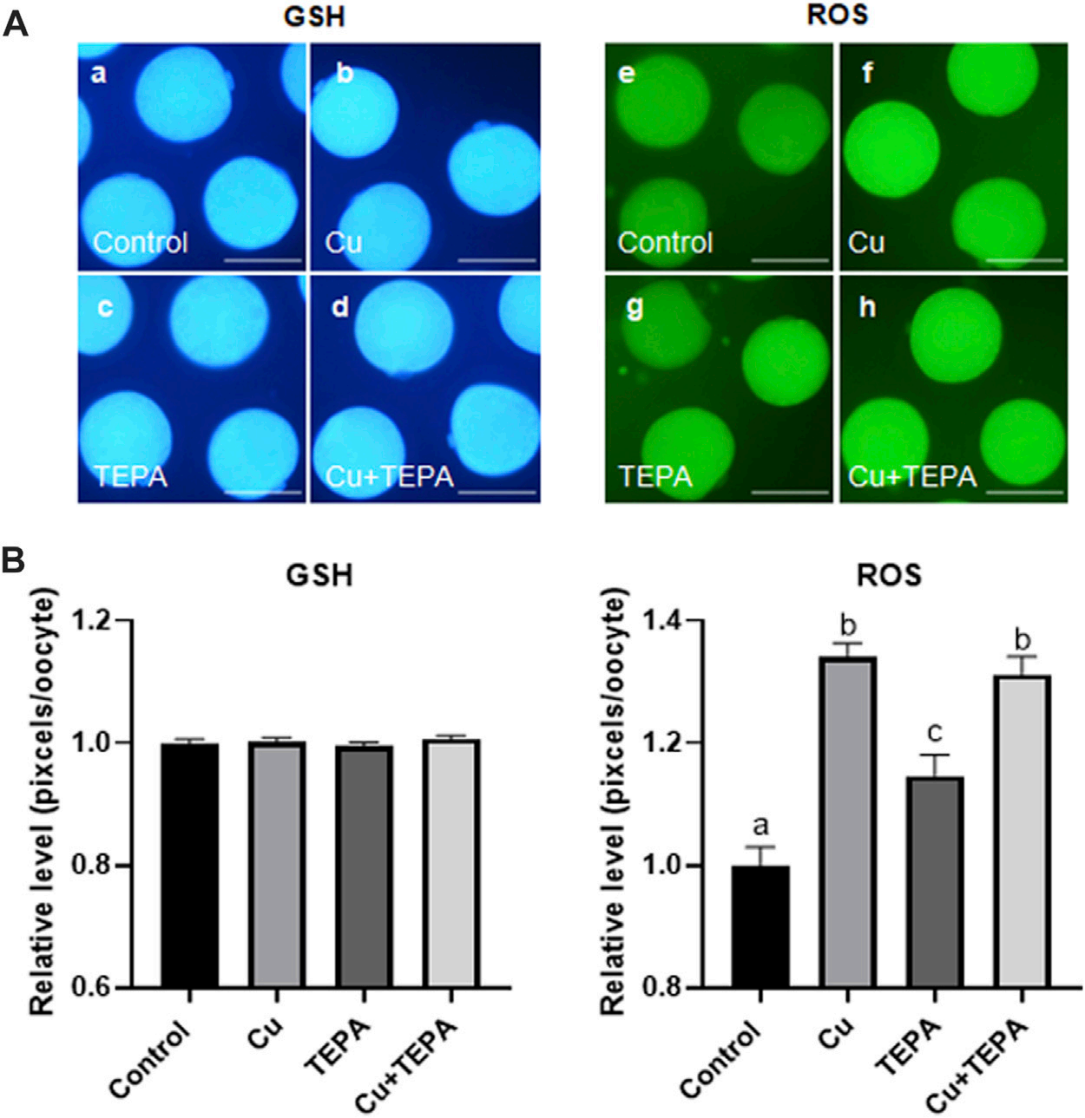
Epifluorescence photomicrographic images of porcine oocytes matured *in vitro*. **(A)** Oocytes were stained using Cell Tracker Blue (a–d) and 2′,7′-dichlorodihydrofluorescein diacetate (e–h) to measure the intracellular levels of glutathione (GSH) and reactive oxygen species (ROS), respectively. Metaphase II (MII) oocytes derived from *in vitro* maturation (IVM) in the presence of Cu or TEPA. Scale bar = 100 μm. **(B)** Effect of Cu supplementation and chelation during IVM on the intracellular GSH and ROS levels in matured porcine oocytes. Within each group (ROS) of endpoints, the bars with different letters (a–c) are significantly different (*p* < 0.05). GSH samples, N = 30; ROS samples, N = 30. For all graphs, the values represent the mean ± SEM. The experiment was replicated three or four times.

To identify the effect of Cu chelation during IVM on cytoplasmic maturation, we examined the intracellular GSH and ROS levels in MII oocytes derived from maturation media supplemented with Cu or/and TEPA. As shown in Figures 4A,B, there were no significant differences in the intracellular GSH levels among groups. However, Cu supplementation and chelation significantly increased the intracellular ROS levels (1.34 ± 0.02 and 1.15 ± 0.03, respectively) compared to those of the control (1.00 ± 0.03). Cu supplementation led to a higher ROS level increase than Cu chelation.

### 3.5 Effect of copper chelation during *in vitro* maturation on the transcript levels of folliculogenesis-related genes

**FIGURE 5:**
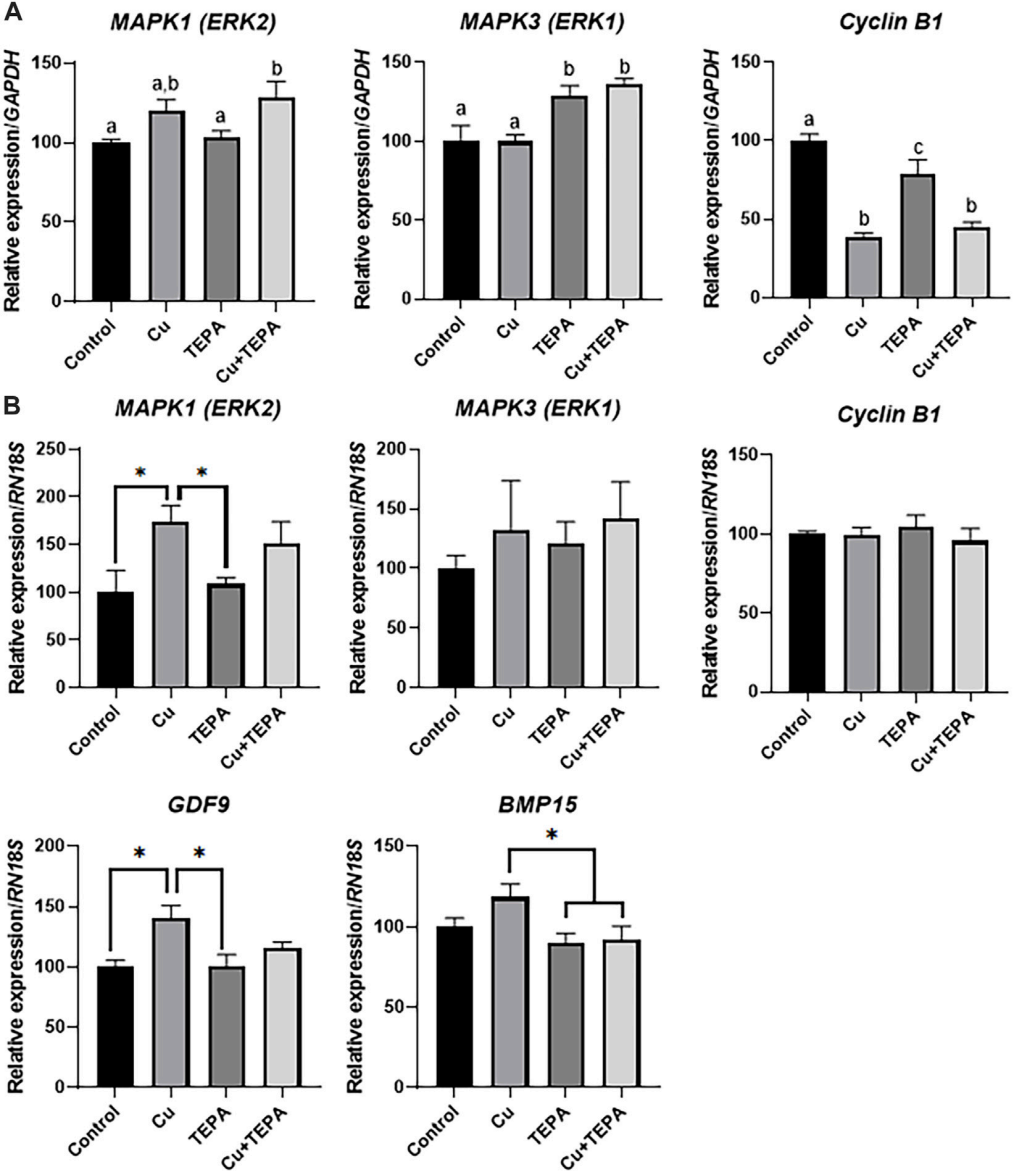
Effect of Cu chelation during *in vitro* maturation (IVM) on the relative mRNA expression levels of folliculogenesis-related genes. **(A)** The relative mRNA expression levels of *MAPK1*, *MAPK3*, and *Cyclin B1*, normalized to the expression levels of *GAPDH*, were compared among the cumulus cells of each group. **(B)** The relative mRNA expression levels of *MAPK1*, *MAPK3*, *Cyclin B1*, *GDF9*, and *BMP15*, normalized to the expression levels of *RN18S*, were compared among oocytes in each group. For all graphs, the values represent the mean ± SEM. The experiment was replicated three times. The values (a–c) with different superscripts within the same column are significantly different (*p* < *0.05*). Asterisks indicate statistical significance (*p < 0.05).

We analyzed the transcript levels of folliculogenesis-related genes according to Cu chelation during IVM. In cumulus cells, Cu chelation with TEPA significantly increased the *MAPK3* (*ERK1*) levels. Cumulus cells derived from COCs matured with Cu and TEPA showed significantly increased levels of the *MAPK1* (*ERK2*) and *MAPK3* (*ERK1*) genes. Interestingly, the level of the *cyclin B1* gene, which is related to the cell cycle, was significantly decreased when COCs were matured using Cu and Cu + TEPA supplementation compared to that of the control (Figure 5A). In the case of oocytes, the *MAPK1* (*ERK2*) and *GDF9* levels were significantly increased in the Cu-treated group compared to those of the control and TEPA-treated groups (Figure 5B). The *BMP15* expression level was significantly increased in the Cu-treated group compared to that of the TEPA and Cu + TEPA groups (Figure 5B).

### 3.6 Effect of copper chelation during *in vitro* maturation on the developmental competence

**FIGURE 6:**
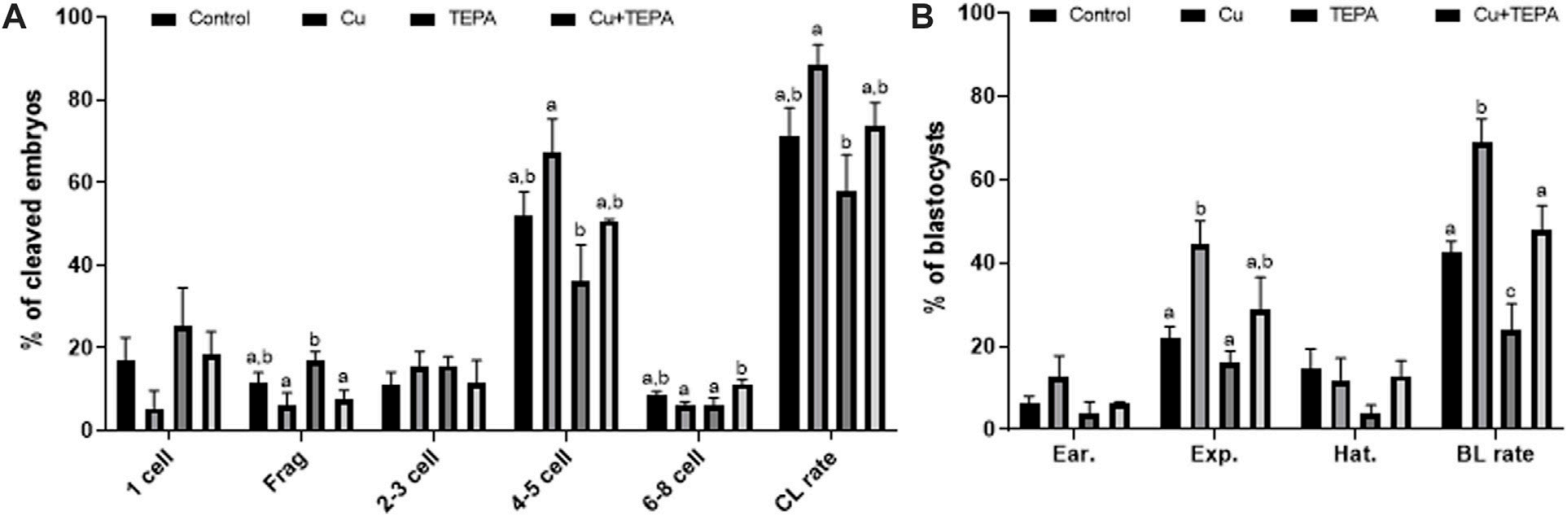
Effect of Cu chelation during *in vitro* maturation (IVM) on the cleavage pattern **(A)** and blastocyst formation pattern **(B)** of the parthenogenetic activation (PA) embryos. Within each endpoint, the bars with different letters (a–c) are significantly different (*p* < 0.05) for various treatment conditions. Frag, Fragmentation; CL, cleavage; BL, blastocyst. The cleavage rate was measured on day 2, and the blastocyst formation rate was evaluated on day 7 of culture. For all graphs, the values represent the mean ± SEM. The experiment was replicated thrice.

We evaluated the effects of Cu chelation during IVM on the subsequent developmental competence of porcine oocytes after PA. There were no significant differences (*p* > 0.05) in the total cleavage rates between the control and the other groups (Figure 6A and Table 2). However, the cleavage pattern of 4–5 cells and the total cleavage rates were significantly decreased in the TEPA-treated group compared to those of the Cu-treated group (Figure 6A). When comparing the blastocyst formation patterns on day 7, the total blastocyst formation rate was significantly lower in the TEPA-treated group than in the control group (Figure 6B and Table 2). Cu treatment significantly increased the blastocyst formation rates (68.9 ± 5.7), whereas the TEPA treatment significantly lowered these (23.8 ± 6.4) compared to those of the control (42.5 ± 2.6) (Table 2). This low blastocyst formation rate obtained using the TEPA treatment was increased to the control level using the Cu treatment (47.9 ± 5.8).

**TABLE 2:** Effect of Cu chelation during *in vitro* maturation (IVM) on the embryonic development after parthenogenetic activation (PA).

Group	No. of embryos Cultured*	No. (%) of embryos developed to		Total cell Number in blastocyst (n)
		≥2-cell	Blastocyst	
Control	129 (71.9 ± 2.0)	92 (71.4 ± 6.6)^a,b^	55 (42.5 ± 2.6)^a^	62.2 ± 3.3^a^ (55)
Cu	151 (83.8 ± 1.3)	133 (88.7 ± 4.7)^a^	103 (68.9 ± 5.7)^b^	63.6 ± 2.5^a,b^ (103)
TEPA	101 (53.0 ± 1.3)	59 (57.9 ± 8.8)^b^	24 (23.8 ± 6.4)^c^	66.3 ± 7.4^a,b^ (24)
Cu + TEPA	142 (82.4 ± 1.8)	105 (73.6 ± 5.7)^a,b^	68 (47.9 ± 5.8)^a^	74.4 ± 3.5^b^ (68)

*: Three times replicated.

n: The number of blastocysts examined.

a Values with different superscripts within a column differ significantly (*p* < 0.05).

b Values with different superscripts within a column differ significantly (*p* < 0.05).

c Values with different superscripts within a column differ significantly (*p* < 0.05).

## 4 Discussion

The CTR1 belongs to the intracellular Cu uptake pathway in the liver and heart tissues, intestinal epithelium, and many other mammalian cells (Ohrvik and Thiele, 2014). In previous studies, the mice with homozygous disruption of the CTR1 locus died during embryogenesis, suggesting that CTR1 is essential for normal embryonic development (Kuo et al., 2001; Lee et al., 2001). The human CTR1 (hCTR1) is localized at the plasma membrane as a homomultimer in mammalian cells (Lee et al., 2002). In bovine oocytes, CTR1 is located at the plasma membrane (Anchordoquy et al., 2017). Klomp et al. (2002) compared the intracellular distribution of hCTR1 according to the cell type and found that hCTR1 was also predominantly located at the plasma membrane in colon carcinoma cell lines. However, hCTR1 was localized in the cytoplasmic vesicular compartment, concentrated around the nucleus, in the cervical carcinoma cell line HeLa. In our immunostaining results (Figure 1), the porcine oocytes showed a similar CTR1 localization to that of the HeLa cell line. As hCTR1 overlapped with the Golgi markers in HeLa cells, it is thought that the CTR1 in porcine oocytes is mainly located in the trans-Golgi network or vesicular compartment. Conversely, in porcine cumulus cells, CTR1 was evenly distributed throughout the cytoplasm. These results suggested that the CTR1 localization may be different for each cell type and species. According to the western blot results, the expression of CTR1 was the highest in mature cumulus cells (Figure 1), which suggests that Cu transport must be actively performed during the maturation of porcine oocytes.

A decrease in the Cu concentration in porcine oocytes was induced by TEPA treatment. TEPA has been used to create functional Cu deficiency in cell cultures (Ding et al., 2011). When this chelator was contained to the culture medium of HL-60 cells, the cellular Cu levels and the activities of two Cu-requiring enzymes, Cu/Zn-superoxide dismutase (Cu/Zn-SOD) and cytochrome c oxidase, were decreased (Percival and Layden-Patrice, 1992). Conversely, when copper and TEPA were simultaneously added to the culture media, the Cu levels and the activities of Cu/Zn-SOD and cytochrome c were recovered to the control levels. Cell growth and viability were not affected by TEPA treatment. Therefore, TEPA only decreases the activity of Cu/Zn-SOD but does not change its protein level. In our study, nuclear maturation was inhibited by TEPA-induced Cu deficiency during IVM. The reduction of the polar body extrusion rate by TEPA could be restored to a control level with Cu supplementation. In other words, Cu deficiency during IVM may suppress the progression of oocyte meiosis by reducing the activity of Cu-requiring enzymes.

The process of cumulus cell expansion is a luteinizing hormone-mediated ovulatory process. In our study, Cu chelation significantly increased the *Has2* mRNA expression levels in cumulus cells more than Cu supplementation. HAS2 regulates the synthesis of hyaluronic acid, the main component of the cumulus cell expansion process (Richards and Pangas, 2010; Yung et al., 2019). The hyaluronan accumulated during cumulus cell expansion binds to CD44 and induces the phosphorylation of the tyrosine residues on Cx43 (Yokoo and Sato, 2011). During cumulus expansion, the hyaluronan-CD44 interaction disrupts the gap junctional communication in COCs and inhibits the cAMP transport from cumulus cells to oocytes. This leads to MPF activation and meiotic resumption in oocytes. However, the CEI analysis in our experiment showed no significant increase in cumulus expansion in the TEPA-treated group. The CEI increase was only found in Cu-treated COCs. These results indicate that the increase in the *Has2* transcript levels by TEPA treatment could not lead to cumulus expansion. On the other hand, the increase in *Has2* and *CD44* gene expression by Cu supplementation consequently contributed to cumulus cell expansion and oocyte meiotic resumption.

GSH is known as a key indicator of cytoplasmic maturation. It plays an important role in regulating protein and DNA synthesis by protecting cells from the toxic effects of ROS and altering their redox state (Zhou et al., 2008). In our study, Cu chelation by 25 μM TEPA treatment during IVM of porcine oocytes did not affect the intracellular GSH levels. On the other hand, both Cu supplementation and chelation increased the intracellular ROS levels. TEPA is able to reduce both the Cu levels and SOD-1 activity in mammalian cells without affecting cell viability (Percival and Layden-Patrice, 1992). TEPA treatment at a concentration of 50 μM had no significant effect on the cellular metabolism and function of human umbilical vein endothelial cells (Wang et al., 2016). Therefore, the increased ROS levels induced by TEPA treatment caused oxidative stress in oocytes by reducing SOD1 activity, whereas the ROS levels increased by Cu supplementation did not cause toxic effects.

The cell-cycle checkpoint involved in the G2/M phase (DNA synthesis completed) is controlled by the cyclin B1/CDK1 complex (Porter and Donoghue, 2003). A decreased cyclin B1 mRNA expression level indicates arrest at the G2/M phase (Luo et al., 2018). The multiple cell cycle stages of compact cumulus cells contrast with those of oocytes (Schuetz et al., 1996). The ovulatory stimulus inhibits cumulus cell proliferation, induces oocyte meiotic maturation, and simultaneously stimulates the secretion of hyaluronic acid, steroids, and prostaglandins. The loss of cumulus cell proliferative activity is closely associated with COC expansion (Schuetz et al., 1996). Therefore, it can be considered that the decrease in the cyclin B1 mRNA level in Cu- or TEPA-treated cumulus cells is related to the increase in COC expansion. However, TEPA-treated cumulus cells showed that the mRNA expression level of ERK1/2, which is known to regulate cyclin B1 expression, was independently regulated. The growth differentiation factor 9 (GDF9) and bone morphogenetic protein 15 (BMP15) are crucial oocyte-secreted factors (Gilchrist et al., 2004; Juengel et al., 2004). They belong to the transforming growth factor-β (TGF-β) superfamily and enhance oocyte developmental competence during *in vitro* maturation by their known effects on CC function (Hussein et al., 2006). The ERK1/2 present in the oocytes is related to the oocyte meiotic resumption and cumulus expansion during folliculogenesis (Kalous et al., 2018). Cu-treated oocytes have higher blastocyst formation rates with increased levels of GDF9 and ERK2 compared to those of the control. Conversely, the TEPA-treated oocytes showed a significantly lower blastocyst formation rate compared to that of the control group without a reduction in the levels of folliculogenesis-related genes. This poor developmental competence can be restored through Cu supplementation.

In conclusion, we identified the localization and relative expression of the Cu transport system in porcine oocytes and cumulus cells. In addition, we showed that Cu chelation by TEPA treatment affects the nuclear maturation of porcine oocytes and their developmental competence *in vitro*, suggesting that Cu is important for meiotic resumption and the developmental competence of porcine oocytes.

## Data Availability

The original contributions presented in the study are included in the article/Supplementary Materials; further inquiries can be directed to the corresponding author.

## Abbreviations

CEI, Cell expansion index; COC, Cumulus-oocyte complexes; IVM, *in vitro* maturation; LH, Luteinizing hormone; PA, Parthenogenetic activation; PBS, Phosphate-buffered saline; ROS, Reactive oxygen species; SEM, Standard error of the mean.
